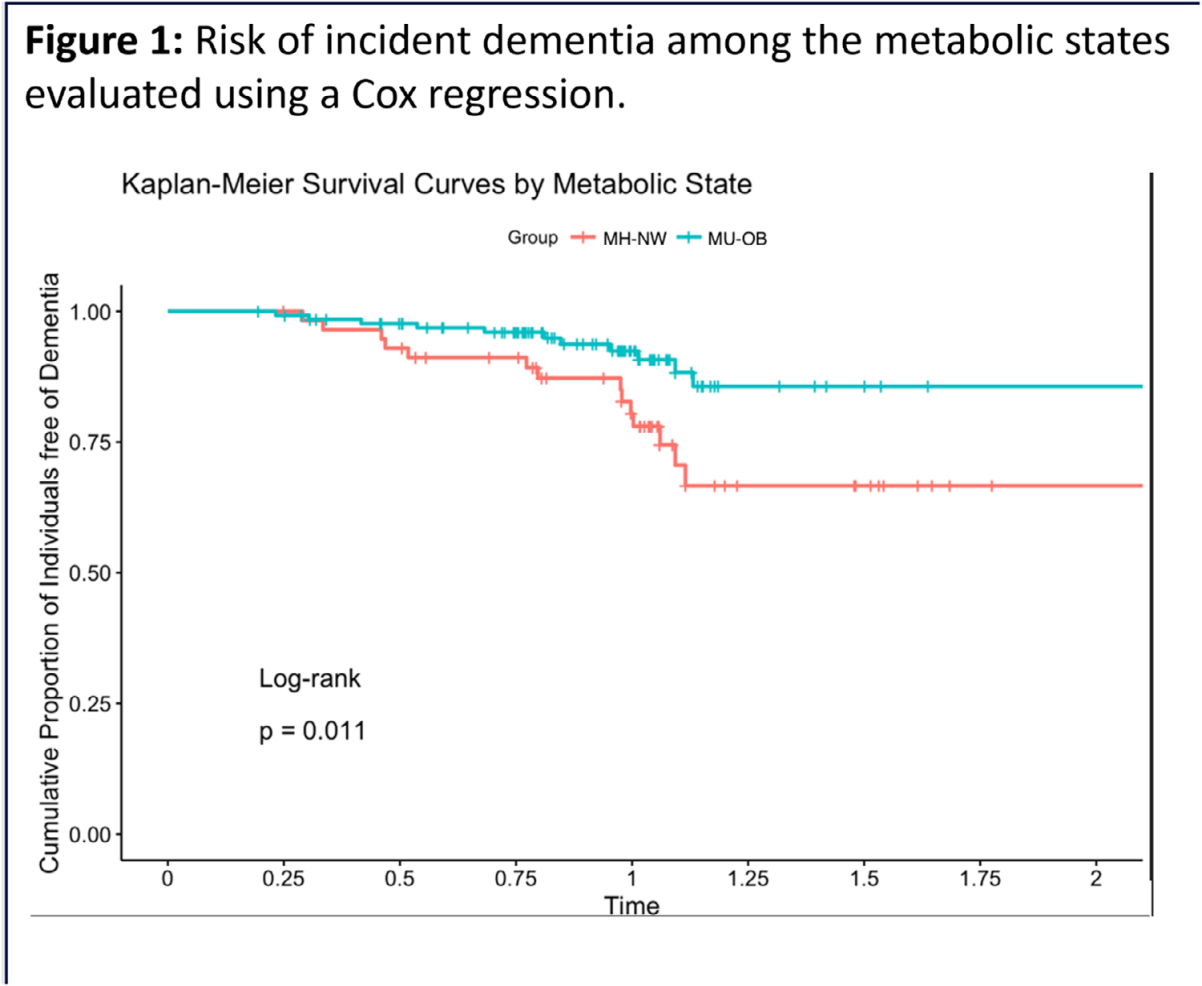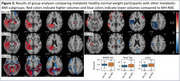# Investigation of Brain Signatures Across Diverse Metabolic States in MCI

**DOI:** 10.1002/alz.086466

**Published:** 2025-01-09

**Authors:** Filippos Anagnostakis, Sindhuja Tirumalai Govindarajan, Elizabeth Mamourian, Guray Erus, Randa Melhem, Yuhan Cui, Ahmed Abdulkadir, Duygu Tosun, Haochang Shou, Ilya M. Nasrallah, Christos Davatzikos

**Affiliations:** ^1^ Artificial Intelligence in Biomedical Imaging Laboratory (AIBIL), Center for and Data Science for Integrated Diagnostics (AI2D), Perelman School of Medicine, University of Pennsylvania, Philadelphia, PA USA; ^2^ University of Pennsylvania, Philadelphia, PA USA; ^3^ Department of Radiology and Biomedical Imaging, University of California, San Francisco, San Francisco, CA USA; ^4^ Centre for Biomedical Image Computing and Analytics, University of Pennsylvania, Philadelphia, PA USA

## Abstract

**Background:**

Late‐life obesity has been reported to have a negative relationship with risk for dementia and has been associated with lower risk of incident Alzheimer’s disease (AD) in non‐demented individuals. However, associations of obesity and cognition solely in patients with mild cognitive impairment (MCI) is unknown. These associations may be confounded by vascular risk that contributes to metabolic syndrome (MetS). This study investigates the complex interplay between obesity, metabolic syndrome, and neurodegeneration in MCI. We analyze neuroimaging measures, AD biomarkers including amyloid‐beta (Aβ) and tau levels in the cerebrospinal fluid (CSF), and a machine‐learning based imaging signature of AD‐like brain atrophy (the SPARE‐AD index) in distinct metabolic subgroups.

**Methods:**

This study included N = 705 MCI patients (age = 75.3+‐7 years, 61% females) scanned with a median follow‐up interval of 30.3 months from the Alzheimer’s Disease Neuroimaging Initiative (ADNI). N = 139 participants developed dementia. Participants were categorized as metabolic healthy (MH, N = 126) or unhealthy (MU, N = 579) based on MetS criteria, and further as normal weight (NW), overweight (OW), or obese (OB) based on their BMI. Risk of incident dementia among the metabolic states was evaluated using Cox regression. Volumetric brain changes, SPARE‐AD, and CSF total‐tau, p‐tau_181_, and Aβ_1‐42_ were analyzed separately using linear models with metabolic state as the predictor, corrected for APOE genotype, age, sex, education, and intracranial volume (volumetric data only). Results were corrected for multiple comparisons using the False Discovery Rate method.

**Results:**

When compared to MH‐NW participants, only MU‐OB participants had a significantly lower risk for incident dementia (Hazard Ratio = 0.430, p<0.05), lower (more normal) SPARE‐AD (p = 0.029), total‐tau (p = 0.02) and p‐tau_181_ (p = 0.006). MU‐OB and MU‐OW participants had significantly higher volumes (p<0.05) of bilateral hippocampi, left temporal white matter, left posterior cingulate gyrus, and the right parahippocampal gyrus, and significantly lower volumes in the right anterior orbital gyrus. A similar pattern of was not observed in other subgroups.

**Conclusion:**

Obese MCI patients with MetS had lower incident dementia, less AD‐like brain shrinkage and more favorable AD biomarkers. Our findings suggest that the cognitive impairment in MU‐high BMI groups might originate from causes unrelated to AD, and might be related to the vascular and metabolic changes.